# Black pus from a worn-out elbow arthroplasty

**DOI:** 10.5194/jbji-7-33-2022

**Published:** 2022-02-07

**Authors:** Marjan Wouthuyzen-Bakker, Alexander L. Boerboom

**Affiliations:** 1 Department of Medical Microbiology and Infection Prevention, University of Groningen, University Medical Center Groningen, Groningen, the Netherlands​​​​​​​; 2 Department of Orthopaedic Surgery, University of Groningen, University Medical Center Groningen, Groningen, the Netherlands

## 1 Clinical picture

**Figure 1:**
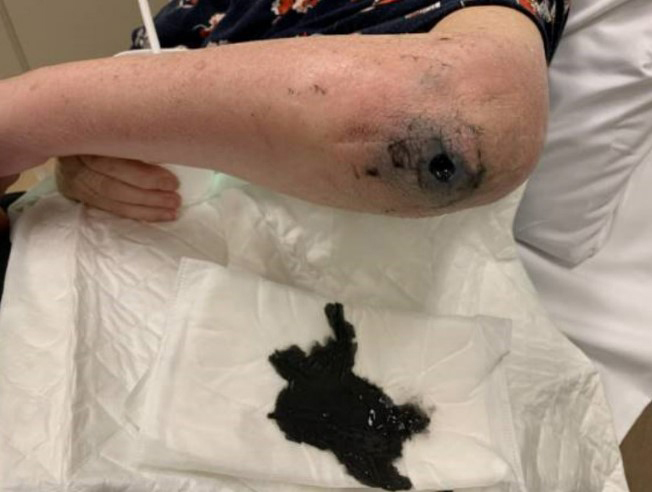
Draining sinus with black pus.

**Figure 2:**
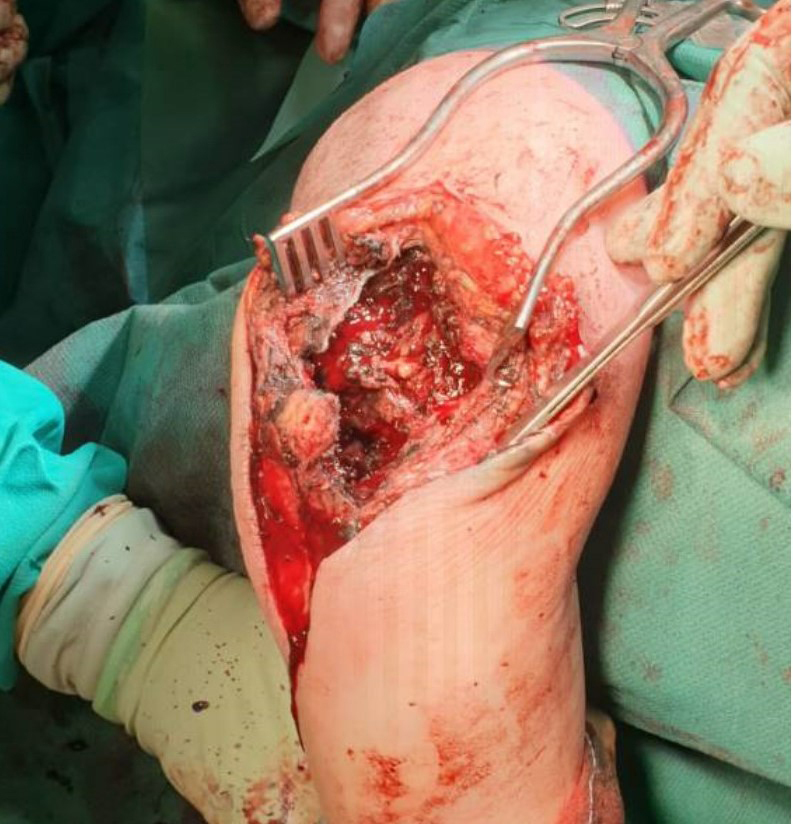
Grey-coloured synovitis and necrosis caused by metallic wear debris due to the release of cobalt, chromium, and titanium particles (Brinkman et al., 2007).

A 64-year-old woman with a medical history of rheumatoid arthritis and multiple joint prostheses presented to our hospital. She had been experiencing pain and a greyish swelling in her left elbow, which had undergone arthroplasty 25 years prior. The swelling eventually opened and drained black metal-like pus (Fig. 1). The prosthesis (a Kudo type 15) was extracted in a two-stage exchange procedure in which dark-grey debris and necrosis were observed (Fig. 2). Cultures revealed the presence of a polymicrobial periprosthetic joint infection (PJI). The patient was treated with amoxicillin and clavulanic acid for 6 weeks and had a flail elbow for 1 year. Finally she received a successful revision arthroplasty with a Coonrad-Morrey elbow prosthesis.

## 2 Conclusion

A worn-out elbow prosthesis causes polyethylene wear and, eventually, severe metallosis when not revised in a timely manner. Damage to the joint, surrounding bone, and soft tissue results in an impaired local immunity and sinus to the skin. These factors make the prosthetic joint more prone to subsequent polymicrobial infection (Barba et al., 2015; Tai et al., 2021).

## Data Availability

No data sets were used in this article.
